# Deterioration of headache impact and health-related quality of life in migraine patients after cessation of preventive treatment with CGRP(−receptor) antibodies

**DOI:** 10.1186/s10194-021-01368-7

**Published:** 2021-12-31

**Authors:** Maria Terhart, Jasper Mecklenburg, Lars Neeb, Lucas Hendrik Overeem, Anke Siebert, Maureen Steinicke, Bianca Raffaelli, Uwe Reuter

**Affiliations:** 1 Department of Neurology, Charité Universitätsmedizin Berlin, Charitéplatz 1, 10117 Berlin, Germany; 2 Universitätsmedizin Greifswald, Greifswald, Germany

## Abstract

**Background:**

Migraine preventive treatment with CGRP(−receptor) monoclonal antibodies (mAbs) has a positive effect on patients’ health-related quality of life (HRQoL). The German treatment guidelines recommend discontinuing successful treatment with CGRP(−receptor) mAbs after 6–12 months. We aimed to evaluate headache-specific and generic HRQoL for three months after discontinuation of CGRP(−receptor) mAb treatment.

**Methods:**

We conducted a prospective, longitudinal cohort study, including patients with migraine after 8–12 months of therapy with a CGRP(−R) mAb and before a planned discontinuation attempt. HRQoL was assessed at the time of the last mAbs injection (V1), eight weeks later (V2), and sixteen weeks later (V3). For headache-specific HRQoL, we used the Headache Impact Test-6 (HIT-6). Generic HRQoL was determined with the EuroQol-5-Dimension-5-Level (ED-5D-5L) form, and the Short-Form 12 (SF-12), which comprises a Physical Component Summary (PCS-12) and a Mental Component Summary (MCS-12).

Questionnaires’ total scores were compared across the three observation points using nonparametric procedures.

**Results:**

The study cohort consisted of *n* = 61 patients (*n* = 29 treated with the CGRP-receptor mAb erenumab and *n* = 32 with the CGRP mAbs galcanezumab or fremanezumab). The HIT-6 sum score was 59.69 ± 6.90 at V1 and increased by 3.69 ± 6.21 at V3 (*p* < 0.001), indicating a greater headache impact on patients’ lives. The mean total EQ-D5-L5 score declined from 0.85 ± 0.17 at V1 by − 0.07 ± 0.18 at V3 (*p* = 0.013). Both Mental and Physical Component Scores of the SF-12 worsened significantly during treatment discontinuation: The PCS-12 total score decreased by − 4.04 ± 7.90 from V1 to V3 (*p* = 0.013) and the MCS-12 score by − 2.73 ± 9.04 (*p* = 0.003). Changes in all questionnaires’ scores but the MCS-12 were already significant in the first month of the drug holiday (V2).

**Conclusions:**

Our results show a significant decline in headache impact and generic HRQoL of migraine patients after treatment discontinuation of a CGRP(−R) mAb. The observed deterioration is above the established minimally clinically important differences for each of the questionnaires and can therefore be considered clinically meaningful. Monitoring HRQoL during a discontinuation attempt could facilitate the decision whether or not to resume preventive treatment with CGRP(−R) mAbs.

## Introduction

Migraine is a disabling disease, which can require prophylactic treatment [1]. The monoclonal antibodies (mAbs) erenumab, galcanezumab and fremanezumab are safe and efficient migraine preventive drugs. They interfere with the calcitonin-gene related peptide (CGRP) pathway to prevent migraine but differ in their exact target. While erenumab targets the CGRP-receptor (CGRP-R), galcanezumab and fremanezumab target the CGRP peptide [2, 3].

In addition to the reduction of migraine frequency and acute medication use, CGRP(−R) mAbs reduce migraine-related disability and increase the quality of life of migraine patients in placebo-controlled randomized clinical trials [4–14]. These observations could be confirmed in real-world studies [15, 16].

Treatment guidelines of the German Migraine and Headache Society (Deutsche Migräne- und Kopfschmerzgesellschaft, DMKG) and the European Headache Federation (EHF) recommend a treatment pause after 6–12 months of successful prophylactic therapy in order to reassess the treatment need [17, 18]. Recent real-world studies describe a worsening of migraine after the discontinuation of CGRP(−R) mAbs [19, 20]. We reported a significant increase in monthly migraine days (MMD) already one month after the discontinuation of CGRP(−R) mAb therapy with a return to baseline levels after three months [21]. To date, no real-world data about health-related quality of life (HRQoL) after the cessation of mAb therapy exists.

HRQoL gained importance in recent years as an outcome in clinical trials for migraine prophylaxis [22–25]. Both headache-specific questionnaires, such as the Headache Impact Test 6 (HIT-6), and generic questionnaires, e.g. the Short Form 12 (SF-12), are recommended to assess HRQoL in migraine patients [24, 25].

Migraine day frequency correlates negatively with HRQoL [26]. However, the burden of migraine on patients is influenced by a host of factors [27]. During the attack-free time, patients’ lives are affected by the fear of the next attack, avoidance of certain activities, and social stigmatization [28, 29]. A focus only on MMD or acute medication use during a discontinuation attempt could be misleading. The assessment of HRQoL changes over time can help to determine the treatment need, which may lead to the resumption of CGRP(−R) mAbs prophylaxis. We therefore studied headache-specific and generic HRQoL changes after a discontinuation attempt of CGRP(−R) prophylactic therapy.

## Methods

### Study design and population

The design of this longitudinal, prospective cohort study has been published in detail elsewhere [21]. In brief, eligible patients were adults with migraine under prophylactic therapy with a CGRP(−R) mAb for at least eight months. All patients were scheduled for a discontinuation attempt, in accordance with the European Headache Federation (EHF) and German treatment guidelines [17, 18]. Patients were on their first CGRP(−R) mAb and had no other concomitant prophylactic medication. We included patients with episodic (EM) and chronic migraine (CM). The diagnosis of migraine was based on the ICHD3-criteria during the year prior to mAb treatment initiation [30].

All patients who met these criteria between January 2020 and December 2020 were asked to participate in the study.

For this analysis, we separated the patients into two groups: 1) patients who received the CGRP-R mAb erenumab (70 or 140 mg subcutaneous (s.c.) per month; receptor group), and 2) patients who received the CGRP mAb galcanezumab (240 mg s.c. loading dose, 120 mg s.c. monthly) or fremanezumab (225 mg s.c. per month; ligand group).

### Study procedures

The study had a duration of 16 weeks and consisted of three consecutive study visits (V1–3). V1 was performed at the time of the last mAb injection. The second visit (V2) was scheduled eight weeks and the third visit (V3) 16 weeks after the last mAb injection.

At each visit, we recorded headache data of the previous month [21]. Additionally, the patients completed the following HRQoL questionnaires: The HIT-6, the SF-12, including its Physical Component Summary (PCS-12), and Mental Component Summary (MCS-12), and the EuroQol-5-Dimension-5-Level (EQ-5D-5L) form.

At V1 and V3, patients documented data in the questionnaires independently on-site, while study staff was available for questions. V2 was performed over the phone and the questionnaires were sent to the patients via mail. We confirmed that patients filled out and sent back the questionnaires within one week. Questionnaires were immediately checked for completeness to avoid missing items.

### Instruments

All used questionnaires are validated for the German language and were analyzed according to the published instructions [31–36].

### Headache-impact-test 6 (HIT-6)

The HIT-6 is a 6-item tool to measure headache impact on a patient’s life during the previous four weeks [37]. It takes six dimensions into account: pain, social functioning, role functioning, vitality, cognitive functioning, and psychological distress [38]. Each item is answered on a 5-point Likert scale as follows: “never” (6 points), “rarely” (8 points), “sometimes” (10 points), “very often” (11 points), and “always” (13 points).

The total HIT-6 score ranges from 36 to 78 points. Scores can be ranked into four categories depending on the headache impact on the patient’s life. HIT-6 scores 36–49 indicate little-to-no impact, 50–55 moderate impact, 56–59 substantial impact, and 60–78 severe impact [32].

### EuroQol-5-Dimension-5-level (EQ-5D-5L)

The EQ-5D-5L assesses patients’ HRQoL on the exact day they are filling out the questionnaire. It allows calculating one single score for patients’ general HRQoL, taking five domains into account: mobility, self-care, usual activities, pain/discomfort, and anxiety/depression [39]. The patients can state the severity grade in every domain as follows: no problems, slight problems, moderate problems, severe problems, ‘unable to’/extreme problems [36].

A response pattern indicating no problems in any of these areas translates to an EQ-5D-5L index score of 1.00. Representative German population samples have shown a mean index score of 0.88 ± 0.18 [40].

### Short form 12 (SF-12)

The SF-12 comprises 12 questions and aims to measure patients’ HRQoL with two scores: the Mental Component Summary (MCS-12) and the Physical Component Summary (PCS-12). The MCS-12 includes questions assessing the patient’s vitality, social functioning, emotional role fulfillment, and mental health. The Physical Component Summary (PCS-12) assesses physical functioning, physical role fulfillment, bodily pain, and general health [33, 34]. The questions refer to the respondent’s condition within the last four weeks. The German average in the SF-12 validation study was 49.60 ± 8.70 for the PCS-12 and 52.30 ± 8.00 for the MCS-12 [33]. Due to the homogeneity of our study population in terms of sex and age, we used the primary scores for the German population as reference values.

Higher PCS and MCS values indicate a better HRQoL.

### Endpoints

Outcomes of the study were the questionnaires’ total scores at each study visit. Primary endpoint was the change in the HIT-6 sum scores across the four-month observation period. Secondary endpoints were the changes in the EQ-5D-5L and SF-12 scores as well as the score changes within the two study groups over all study visits.

In a subgroup analysis, we assessed the changes in questionnaires’ total scores between V1 and V3 in patients who did not experience a clinically relevant worsening of MMD in this period. We defined a 30% or higher worsening as clinically relevant following international guidelines for trials of prophylactic treatment in chronic migraine [24].

### Statistical analysis

We used SPSS 27 (IBM, NY, USA) to perform all statistical analyses. Descriptive statistics were performed to assess demographic and anamnestic data using mean ± standard deviation for numeric variables, and absolute and percentage frequencies for categorical variables. Outcomes were tested for normal distribution using the Kolmogorov-Smirnov test. Because this test revealed a non-normal distribution, we used the following non-parametric tests: the Friedman test with post-hoc pairwise comparisons for dependent samples or the Mann-Whitney U test for independent samples.

A two-tailed *p*-value < 0.05 was considered statistically significant. *P*-values were adjusted for multiple comparisons using the Bonferroni method.

### Ethics

The study was approved by the Charité Ethical Committee (EA1/274/19). All participants gave written informed consent after they received adequate study information.

## Results

### Demographics and patients’ characteristics

Between January 2020 and December 2020, *n* = 65 patients met the criteria for study inclusion, and *n* = 63 (96.9%) agreed to participate. A total of *n* = 61 patients (*n* = 29 receptor group and *n* = 32 ligand group) provided complete data sets.

Demographic and anamnestic data were evenly distributed between the groups (Table 1, *p* > 0.28, n.s.). Demographic characteristics of the cohort and the evolution of headache days have been published in detail elsewhere [21].

**Table 1:** Basic demographic and anamnestic data as well as questionnaire scores at V1

	All Patients	CGRP-receptor mAb-group	CGRP mAb group
N	61	29	32
Age (years)	49.97 ± 11.28	50.55 ± 12.82	49.44 ± 9.86
Sex (female)	59 (96.7%)	28 (96.6%)	31 (96.1%)
Chronic migraine	40 (65.6%)	17 (58.6%)	23 (71.9%)
Months of treatment before discontinuation	9.64 ± 1.14	9.69 ± 1.37	9.59 ± 0.91
MMD	8.59 ± 6.60	9.41 ± 5.92	7.84 ± 7.17

Data for all patients and both subgroups (CGRP-receptor mAb and CGRP mAb group). Data expressed as mean ± standard deviation or absolute number (%). MMD = monthly migraine days.

### Migraine evolution after treatment discontinuation

MMDs increased from 8.59 ± 6.60 at V1 to 12.84 ± 6.86 in the fourth month after cessation of therapy (V3). This represents an increase to a similar MMD level as before mAb therapy initiation, which was 13.52 ± 6.34.

Significantly more patients with the initial diagnosis of CM prior to mAb treatment met the criteria for CM also at V3 (57.5% of patients with prior CM vs. 21.7% of patients with prior EM, *p* = 0.012 as measured with chi-squared test).

The subgroup of patients with a less than 30% increase of MMD between V1 and V3 consisted of *n* = 26 women (42.6%) with an age of 50.92 ± 12.73 years. Most of them had chronic migraine (*n* = 17, 65.4%) with an average of 14.54 ± 7.03 MMD prior to treatment begin.

### Changes headache impact

**Fig. 1:**
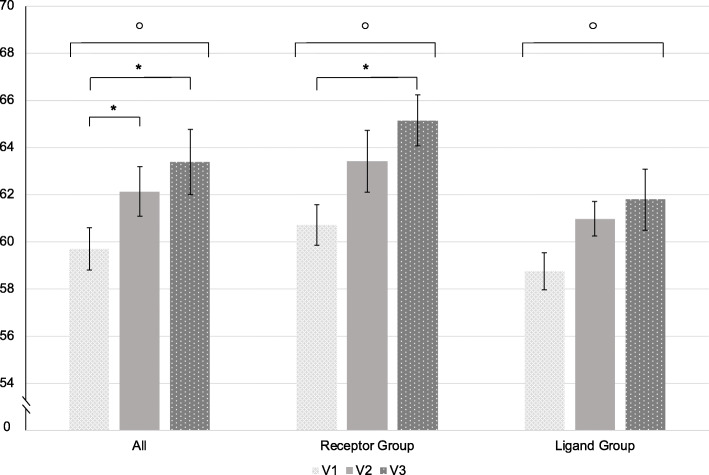
Mean HIT-6 sum scores over all patients, in the receptor group and the ligand group. Values are illustrated as mean ± standard error. _o_ = statistically significant in the Friedman test (*p* < 0.05). * = statistically significant in post-hoc pairwise comparisons with Bonferroni correction (*p* < 0.05). V1 = time of last injection; V2 = 8 weeks after last injection; V3 = 16 weeks after the last injection

The HIT-6 sum scores deteriorated significantly over time (*p* < 0.001) (Table 2, Fig. 1).

**Table 2:** Mean total scores of all questionnaires and the differences between the time points

	V1	V2	V3	Difference V1-V2	Difference V1-V3	p-values
**HIT-6** All Patients	59.69 ± 6.90	62.13 ± 6.68	63.38 ± 6.16	2.44 ± 6.98	3.69 ± 6.21	*p* < 0.001 V1 vs. V2 *p* = 0.006 V1 vs. V3 *p* < 0.001 V2 vs. V3 *p* = 0.568
**HIT-6** CGRP-receptor mAb group	60.72 ± 5.61	63.41 ± 7.14	65.14 ± 4.04	2.69 ± 6.95	4.41 ± 6.01	*p* < 0.001 V1 vs. V2 *p* = 0.091 V1 vs. V3 *p* < 0.001 V2 vs. V3 *p* = 0.229
**HIT-6** CGRP mAb group	58.75 ± 7.85	60.97 ± 6.11	61.78 ± 7.29	2.22 ± 7.11	3.03 ± 6.42	*p* = 0.022 V1 vs. V2 *p* = 0.086 V1 vs. V3 *p* = 0.062 V2 vs. V3 *p* > 0.999
**EQ-5D-5L** All Patients	0.85 ± 0.17	0.77 ± 0.18	0.77 ± 0.20	−0.07 ± 0.19	−0.07 ± 0.18	*p* = 0.001 V1 vs. V2 *p* = 0.008 V1 vs. V3 *p* = 0.013 V2 vs. V3 *p* > 0.999
**EQ-5D-5L** CGRP-receptor mAb group	0.85 ± 0.18	0.77 ± 0.17	0.77 ± 0.19	−0.08 ± 0.21	−0.09 ± 0.18	*p* < 0.001 V1 vs. V2 *p* = 0.002 V1 vs. V3 *p* = 0.002 V2 vs. V3 *p* > 0.999
**EQ-5D-5L** CGRP mAb group	0.84 ± 0.16	0.77 ± 0.20	0.78 ± 0.21	−0.07 ± 0.17	−0.06 ± 0.18	*p* = 0.485
**PCS-12** All Patients	39.18 ± 9.70	35.74 ± 9.02	35.14 ± 8.98	−3.44 ± 8.39	−4.04 ± 7.90	*p* = 0.005 V1 vs. V2 *p* = 0.030 V1 vs. V3 *p* = 0.013 V2 vs. V3 *p* > 0.999
**PCS-12** CGRP-receptor mAb group	38.43 ± 9.06	35.07 ± 8.41	33.64 ± 8.06	− 3.36 ± 7.94	−4.79 ± 7.00	*p* = 0.002 V1 vs. V2 *p* = 0.31 V1 vs. V3 *p* = 0.005 V2 vs. V3 *p* > 0.999
**PCS-12** CGRP mAb group	39.86 ± 10.34	36.35 ± 9.63	36.50 ± 9.67	− 3.51 ± 8.90	−3.36 ± 8.69	*p* = 0.464
**MCS-12** All Patients	43.95 ± 10.33	41.44 ± 11.43	41.22 ± 11.21	−2.51 ± 8.46	−2.73 ± 9.04	*p* = 0.003 V1 vs. V2 *p* = 0.089 V1 vs. V3 *p* = 0.003 V2 vs. V3 *p* = 0.832
**MCS-12** CGRP-receptor mAb group	44.26 ± 9.85	40.80 ± 11.83	40.42 ± 10.27	−3.46 ± 8.13	−3.83 ± 8.33	*p* = 0.066
**MCS-12** CGRP mAb group	43.68 ± 10.90	42.02 ± 11.20	41.95 ± 12.13	−1.66 ± 8.79	−1.73 ± 9.66	*p* = 0.041 V1 vs. V2 *p* = 0.401 V1 vs. V3 *p* = 0.044 V2 vs. V3 > 0.999

Data for all patients and both subgroups (CGRP-receptor mAb and CGRP mAb group). Data is expressed as mean ± standard deviation. The first *p*-value stated is derived from the Friedman test. The following *p*-values are derived from post-hoc pairwise comparisons with Bonferroni correction. V1 = time of last injection, V2 = 8 weeks after last injection, V3 = 16 weeks after last injection.

Over half of patients (*n* = 34, 54.1%) reported a worsening of ≥2.5 points and over one-third (*n* = 23, 37.7%) a worsening of ≥6 points from V1 to V3.

Subgroup analyses revealed a worsening in both the receptor and the ligand group over all time points (Fig. 1).

There was a positive correlation between the increase in MMD after treatment cessation and the deterioration of the HIT-6 scores (*p* = 0.014, r = 0.313). However, the HIT-6 sum scores increased significantly from 60.00 ± 7.56 to 62.96 ± 7.21 also in patients with a less than 30% increase in MMD (*p* = 0.011).

### Changes in generic HRQoL

#### Effects of treatment discontinuation on the EQ-D5-L5

**Fig. 2:**
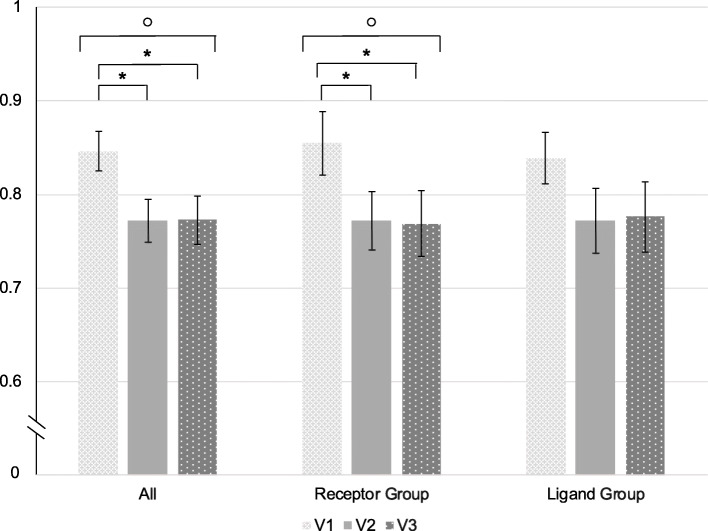
EQ-5D-5L index scores over all patients, in the receptor group and the ligand group. Values are illustrated as mean ± standard error. _o_ = statistically significant in the Friedman test (*p* < 0.05). * = statistically significant in post-hoc pairwise comparisons with Bonferroni correction (*p* < 0.05). V1 = time of last injection; V2 = 8 weeks after last injection; V3 = 16 weeks after the last injection

The mean total EQ-D5-L5 score worsened significantly during the treatment pause (*p* < 0.001) (Table 2, Fig. 2). The changes were only significant in patients treated with a CGRP-R mAb (*p* < 0.001). Changes in the EQ-5D-5L score did not correlate with changes in MMD from V1 to V3 (*p* = 0.377, r = 0.115).

The subgroup of patients with a less than 30% increase in MMD after mAb cessation also showed a significant decline in EQ-5D-5L values over time (∆ -0.10 points V1 vs. V3, *p* = 0.005).

#### Effects of treatment discontinuation on the short-form (SF-12)

The mental (MCS-12) and physical (PCS-12) components of the SF-12 deteriorated significantly throughout the discontinuation attempt (Table 2). In the subgroup analyses, statistical significance was reached only in the CGRP-R group for the PCS-12 score (*p* = 0.002) and in the ligand group for the MCS-12 score (*p* = 0.041). Neither the PCS-12 nor the MCS-12 score changes between V1 and V3 correlated significantly with the changes in migraine frequency after treatment discontinuation (PCS-12: *p* = 0,296, *r* = 0,136, MCS-12: *p* = 0,186, r = 0,171).

Patients with less than 30% increase of MMD reported a significant decline in PCS-12 total scores from 37.63 ± 10.02 at V1 to 33.53 ± 9.52 at V3 (*p* = 0.021).

## Discussion

This analysis revealed a deterioration in the quality of life of patients with migraine after the cessation of preventive treatment with a CGRP(−R) mAb. The impact of headache on patients’ lives increased and the general wellbeing deteriorated during the three-month medication discontinuation period.

Data on HRQoL after long-term therapy with mAbs and a subsequent medication pause under real world conditions does not exist. The changes in HRQoL in this study are clinically meaningful when compared to established minimally clinically important differences (MCID) for each of the questionnaires. The MCID for the HIT-6 questionnaire can vary between different headache types and populations [41]. For migraine, Smelt et al. determined within-person changes of more than 2.5 points to be meaningful for the clinical practice [41]. Both study groups as well as the subgroup with less than 30% increase in MMD met this threshold in our analysis. MCID for generic HRQoL (EQ-5D-5L and SF-12) in headache patients have not been established yet. However, the threshold for relevant changes has been determined for several chronic diseases (e.g. chronic obstructive pulmonary disease) and pain conditions (e.g. osteoarthritis, low back pain) [42–45]. In these previous studies, the MCID for the EQ-5D-5L ranked between 0.04 and 0.32 and for both SF-12 scale scores between 3.3 and 3.8 [42–45]. The changes in our cohort are well in the range that is considered clinically meaningful in the aforementioned conditions [42–45].

Of note, there was no correlation between the changes in the generic HRQoL questionnaire scores and the change of migraine frequency. Our findings support previous studies, which illustrated that headache frequency is not the only factor influencing HRQoL in patients with migraine [28, 29].

Headache impact was an endpoint in several clinical trials for CGRP(−R) mAbs in migraine preventive treatment. In the double-blind, placebo-controlled trial of erenumab in patients with CM, HIT-6 scores decreased from ~ 63 points at baseline to ~ 57 points after three treatment months [4]. In the STRIVE trial for EM, erenumab led to a HIT-6 reduction from ~ 60 to ~ 54 points in months 4–6 [5]. Similarly, patients with CM treated with fremanezumab reported a decrease from ~ 64 to ~ 57 points at the end of a 12-week double-blind phase [46].

In our cohort, almost 40% of patients reported an increase of ≥6 points in the HIT-6 sum score. The magnitude of change in these patients is comparable to the results of randomized-controlled trials, though in the opposite direction.

The deterioration of HRQoL after mAb cessation is new and expands our knowledge on discontinuation attempts in clinical practice. In the parent study of this analysis, we described an increase of migraine and headache frequency over three months [21]. Acute medication intake went gradually back to baseline levels from before the initiation of mAbs preventative treatment [21]. Two other real-world studies on migraine frequency also found a significant deterioration one and three months after treatment discontinuation [19, 20].

From phase 3 clinical trials, HRQoL data after treatment termination is available for galcanezumab in the prophylaxis of episodic migraine [47, 48]. In both EVOLVE trials, HRQoL was measured with the Migraine-Specific Quality of Life Questionnaire (MSQ) Role Function-Restrictive Domain up to three months after the end of the double-blind treatment phase, when patients received no drug [49]. The initial benefit of galcanezumab on MSQ scores decreased after treatment cessation. Three months after discontinuation, the MSQ scores of patients in the galcanezumab group were not different from the placebo group anymore [49]. The data from our study support the findings from the EVOLVE trials.

This study revealed some differences between patients treated with a CGRP-R mAb and patients treated with a CGRP mAb. For all questionnaires but the MCS-12, HRQoL was more severely impaired three months after the cessation of erenumab than galcanezumab or fremanezumab. This might be due to erenumab’s shorter elimination half-life time with about 21 days [50] as opposed to the half life time of galcanezumab (t/2 = 27 days) and fremanezumab (t/2 = 30 days) [51, 52].

Considering the deterioration of not only headache frequency but also HRQoL during a discontinuation attempt, the question remains, if treatment pauses should be carried out rigorously. It is also matter of debate when to resume mAb prophylaxis if migraine deteriorates. To date, migraine frequency is the main criterion for the prescription of migraine preventative treatment [53]. A steep increase of MMD is likely to influence the physician towards restarting the treatment after a discontinuation attempt. Our study shows that most patients suffer from an impaired HRQoL during treatment discontinuation, even without a relevant worsening of migraine frequency. Monitoring HRQoL could therefore represent an important addition to the evaluation of headache data in clinical practice.

We did not assess the patients’ HRQoL before the start of mAb prophylaxis. Comparison to baseline levels prior to treatment begin could provide further insight into the impact of CGRP(−R) mAbs on the patients’ wellbeing and should be included in further research. Our results might be influenced by the nocebo effect after cessation of a successful treatment. Psychological factors, such as fear of migraine worsening, anxiety or catastrophizing thoughts, might have contributed to the worsening of HRQoL. Further studies should aim to better evaluate such psychological constructs in the context of treatment discontinuation.

This bias cannot be separated from the underlying worsening due to treatment discontinuation in this real-world setting. However, we aimed to provide a real-world picture of treatment discontinuations and such bias is intrinsic in clinical practice.

## Conclusion

Our results show a significant decline in headache impact and generic HRQoL of migraine patients after treatment discontinuation of a CGRP(−R) mAb. The monitoring of HRQoL during a discontinuation attempt provides information in addition to headache diaries and may help to decide if and when to resume preventive therapy.

## Data Availability

The datasets used and/or analyzed during the current study are available from the corresponding author on reasonable request.

## Abbreviations

CGRP: calcitonin gene-related peptide
CGRP-R: calcitonin gene-related peptide receptor
CM: chronic migraine
DMKG: Deutsche Migräne- und Kopfschmerzgesellschaft, German Migraine and Headache Society
EHF: European Headache Federation
EM: episodic migraine
EQ-5D-5L: EuroQoL-5-Dimension-5-Level
HIT-6: Headache Impact Test 6
HRQOL: health-related quality of life
mAbs: monoclonal antibodies
MCID: minimal clinically important differences
MCS-12: Mental Component Summary 12
MMD: Monthly Migraine Days
MSQ: Migraine-Specific Quality of Life Questionnaire
PCS-12: Physical Component Summary 12
SF-12: Short Form 12
t/2: half life time
V1: Study visit 1
V2: Study visit 2
V3: Study visit 3

## References

[1] Lipton RB, Bigal ME, Diamond M, Freitag F, Reed ML, Stewart WF (2007). Migraine prevalence, disease burden, and the need for preventive therapy. Neurology..

[2] Raffaelli B, Neeb L, Reuter U (2019). Monoclonal antibodies for the prevention of migraine. Expert Opin Biol Ther.

[3] Schoenen J, Manise M, Nonis R, Gérard P, Timmermans G (2020). Monoclonal antibodies blocking CGRP transmission: an update on their added value in migraine prevention. Rev Neurol (Paris).

[4] Lipton RB, Tepper SJ, Reuter U, Silberstein S, Stewart WF, Nilsen J, Leonardi DK, Desai P, Cheng S, Mikol DD, Lenz R (2019). Erenumab in chronic migraine: patient-reported outcomes in a randomized double-blind study. Neurology..

[5] Buse DC, Lipton RB, Hallström Y, Reuter U, Tepper SJ, Zhang F, Sapra S, Picard H, Mikol DD, Lenz RA (2018). Migraine-related disability, impact, and health-related quality of life among patients with episodic migraine receiving preventive treatment with erenumab. Cephalalgia..

[6] Ford J, Tassorelli C, Leroux E, Wang S, Ayer D, Nichols R, Detke H (2021). Changes in patient functioning and disability: results from a phase 3, double-blind, randomized, placebo-controlled clinical trial evaluating galcanezumab for chronic migraine prevention (REGAIN). Qual Life Res.

[7] Vernieri F, Altamura C, Brunelli N, Costa CM, Aurilia C, Egeo G (2021). Galcanezumab for the prevention of high frequency episodic and chronic migraine in real life in Italy: a multicenter prospective cohort study (the GARLIT study). J Headache Pain..

[8] Ayer DW, Skljarevski V, Ford JH, Nyhuis AW, Lipton RB, Aurora SK (2018). Measures of functioning in patients with episodic migraine: findings from a double-blind, randomized, placebo-controlled phase 2b trial with Galcanezumab. Headache..

[9] Dodick DW, Silberstein SD, Bigal ME, Yeung PP, Goadsby PJ, Blankenbiller T, Grozinski-Wolff M, Yang R, Ma Y, Aycardi E (2018). Effect of Fremanezumab compared with placebo for prevention of episodic migraine: a randomized clinical trial. Jama..

[10] Lipton RB, Cohen JM, Gandhi SK, Yang R, Yeung PP, Buse DC (2020). Effect of fremanezumab on quality of life and productivity in patients with chronic migraine. Neurology..

[11] Buse DC, Gandhi SK, Cohen JM, Ramirez-Campos V, Cloud B, Yang R, Cowan RP (2020). Improvements across a range of patient-reported domains with fremanezumab treatment: results from a patient survey study. J Headache Pain..

[12] Bigal ME, Dodick DW, Rapoport AM, Silberstein SD, Ma Y, Yang R, Loupe PS, Burstein R, Newman LC, Lipton RB (2015). Safety, tolerability, and efficacy of TEV-48125 for preventive treatment of high-frequency episodic migraine: a multicentre, randomised, double-blind, placebo-controlled, phase 2b study. Lancet Neurol.

[13] Spierings ELH, Ning X, Ramirez Campos V, Cohen JM, Barash S, Buse DC (2021). Improvements in quality of life and work productivity with up to 6 months of fremanezumab treatment in patients with episodic and chronic migraine and documented inadequate response to 2 to 4 classes of migraine-preventive medications in the phase 3b FOCUS study. Headache..

[14] Silberstein SD, Cohen JM, Seminerio MJ, Yang R, Ashina S, Katsarava Z (2020). The impact of fremanezumab on medication overuse in patients with chronic migraine: subgroup analysis of the HALO CM study. J Headache Pain..

[15] Russo A, Silvestro M, Scotto di Clemente F, Trojsi F, Bisecco A, Bonavita S (2020). Multidimensional assessment of the effects of erenumab in chronic migraine patients with previous unsuccessful preventive treatments: a comprehensive real-world experience. J Headache Pain.

[16] Talbot J, Stuckey R, Crawford L, Weatherby S, Mullin S (2021). Improvements in pain, medication use and quality of life in onabotulinumtoxinA-resistant chronic migraine patients following erenumab treatment - real world outcomes. J Headache Pain..

[17] Sacco S, Bendtsen L, Ashina M, Reuter U, Terwindt G, Mitsikostas DD, Martelletti P (2019). European headache federation guideline on the use of monoclonal antibodies acting on the calcitonin gene related peptide or its receptor for migraine prevention. J Headache Pain.

[18] Diener HC, Förderreuther S, Gaul C, Giese F, Hamann T, Holle-Lee D (2020). Prevention of migraine with monoclonal antibodies against CGRP or the CGRP receptor: addition to the S1 guideline: therapy of migraine attacks and prevention of migraine. Recommendations of the Germany society of neurology and the German migraine and headache society. Neurol Res Pract.

[19] Gantenbein AR, Agosti R, Gobbi C, Flügel D, Schankin CJ, Viceic D, Zecca C, Pohl H (2021). Impact on monthly migraine days of discontinuing anti-CGRP antibodies after one year of treatment - a real-life cohort study. Cephalalgia..

[20] De Matteis E, Affaitati G, Frattale I, Caponnetto V, Pistoia F, Giamberardino MA (2021). Early outcomes of migraine after erenumab discontinuation: data from a real-life setting. Neurol Sci.

[21] Raffaelli B, Terhart M, Overeem LH, Mecklenburg J, Neeb L, Steinicke M, Reuter U (2021). Migraine evolution after the cessation of CGRP(−receptor) antibody prophylaxis: a prospective, longitudinal cohort study. Cephalalgia..

[22] Solomon GD (1997). Evolution of the measurement of quality of life in migraine. Neurology..

[23] Becker WJ (2002). Assessing health-related quality of life in patients with migraine. Can J Neurol Sci.

[24] Tassorelli C, Diener HC, Dodick DW, Silberstein SD, Lipton RB, Ashina M, Becker WJ, Ferrari MD, Goadsby PJ, Pozo-Rosich P, Wang SJ, for the International Headache Society Clinical Trials Standing Committee (2018). Guidelines of the international headache society for controlled trials of preventive treatment of chronic migraine in adults. Cephalalgia..

[25] Diener HC, Tassorelli C, Dodick DW, Silberstein SD, Lipton RB, Ashina M, Becker WJ, Ferrari MD, Goadsby PJ, Pozo-Rosich P, Wang SJ, Houle TT, Hoek TC, Martinelli D, Terwindt GM, on behalf of the International Headache Society Clinical Trials Committee (2020). Guidelines of the international headache society for controlled trials of preventive treatment of migraine attacks in episodic migraine in adults. Cephalalgia..

[26] Ruscheweyh R, Müller M, Blum B, Straube A (2014). Correlation of headache frequency and psychosocial impairment in migraine: a cross-sectional study. Headache..

[27] Leonardi M, Raggi A (2019). A narrative review on the burden of migraine: when the burden is the impact on people's life. J Headache Pain..

[28] Lampl C, Thomas H, Stovner LJ, Tassorelli C, Katsarava Z, Laínez JM, Lantéri-Minet M, Rastenyte D, Ruiz de la Torre E, Andrée C, Steiner TJ (2016). Interictal burden attributable to episodic headache: findings from the Eurolight project. J Headache Pain..

[29] Dahlöf CG, Dimenäs E (1995). Migraine patients experience poorer subjective well-being/quality of life even between attacks. Cephalalgia..

[30] Headache Classification Committee of the International Headache Society (IHS) The International Classification of Headache Disorders, 3rd edition. Cephalalgia. 2018;38(1):1–211 10.1177/0333102417738202 29368949

[31] Martin M, Blaisdell B, Kwong JW, Bjorner JB (2004). The short-form headache impact test (HIT-6) was psychometrically equivalent in nine languages. J Clin Epidemiol.

[32] Bayliss M, Batenhorst A (2002). The HIT-6™ a user’s guide.

[33] Gandek B, Ware JE, Aaronson NK, Apolone G, Bjorner JB, Brazier JE, Bullinger M, Kaasa S, Leplege A, Prieto L, Sullivan M (1998). Cross-validation of item selection and scoring for the SF-12 health survey in nine countries: results from the IQOLA project. International quality of life assessment. J Clin Epidemiol.

[34] Kosinski M, Ware JE, Turner-Bowker DM, Gandek B (2007). User's manual for the SF-12v2 health survey : with a supplement documenting the SF-12® health survey.

[35] Ludwig K, von der Schulenburg JM G, Greiner W (2018). German Value Set for the EQ-5D-5L. Pharmacoeconomics..

[36] EuroQol Research Foundation. EQ-5D-5L User Guide, 2019. Available from: https://euroqol.org/publications/user-guides

[37] Kosinski M, Bayliss MS, Bjorner JB, Ware JE, Garber WH, Batenhorst A (2003). A six-item short-form survey for measuring headache impact: the HIT-6. Qual Life Res.

[38] Shin HE, Park JW, Kim YI, Lee KS (2008). Headache impact Test-6 (HIT-6) scores for migraine patients: their relation to disability as measured from a headache diary. J Clin Neurol.

[39] EuroQol Group. EuroQol--a new facility for the measurement of health-related quality of life. Health Policy 1990;16(3):199–208. doi: 10.1016/0168-8510(90)90421-9. PMID: 10109801 10.1016/0168-8510(90)90421-9 10109801

[40] Grochtdreis T, Dams J, König HH, Konnopka A (2019). Health-related quality of life measured with the EQ-5D-5L: estimation of normative index values based on a representative German population sample and value set. Eur J Health Econ.

[41] Smelt AF, Assendelft WJ, Terwee CB, Ferrari MD, Blom JW (2014). What is a clinically relevant change on the HIT-6 questionnaire? An estimation in a primary-care population of migraine patients. Cephalalgia..

[42] Bilbao A, García-Pérez L, Arenaza JC, García I, Ariza-Cardiel G, Trujillo-Martín E, Forjaz MJ, Martín-Fernández J (2018). Psychometric properties of the EQ-5D-5L in patients with hip or knee osteoarthritis: reliability, validity and responsiveness. Qual Life Res.

[43] Hu X, Jing M, Zhang M, Yang P, Yan X (2020). Responsiveness and minimal clinically important difference of the EQ-5D-5L in cervical intraepithelial neoplasia: a longitudinal study. Health Qual Life Outcomes.

[44] Nolan CM, Longworth L, Lord J, Canavan JL, Jones SE, Kon SS (2016). The EQ-5D-5L health status questionnaire in COPD: validity, responsiveness and minimum important difference. Thorax..

[45] Díaz-Arribas MJ, Fernández-Serrano M, Royuela A, Kovacs FM, Gallego-Izquierdo T, Ramos-Sánchez M, Llorca-Palomera R, Pardo-Hervás P, Martín-Pariente OS (2017). Minimal clinically important difference in quality of life for patients with low Back pain. Spine (Phila Pa 1976).

[46] Silberstein SD, Dodick DW, Bigal ME, Yeung PP, Goadsby PJ, Blankenbiller T, Grozinski-Wolff M, Yang R, Ma Y, Aycardi E (2017) Fremanezumab for the preventive treatment of chronic migraine. N Engl J Med 377(22):2113–2122. 10.1056/NEJMoa1709038 10.1056/NEJMoa1709038 29171818

[47] Stauffer VL, Dodick DW, Zhang Q, Carter JN, Ailani J, Conley RR (2018). Evaluation of Galcanezumab for the prevention of episodic migraine: the EVOLVE-1 randomized clinical trial. JAMA Neurol.

[48] Skljarevski V, Matharu M, Millen BA, Ossipov MH, Kim BK, Yang JY (2018). Efficacy and safety of galcanezumab for the prevention of episodic migraine: results of the EVOLVE-2 phase 3 randomized controlled clinical trial. Cephalalgia..

[49] Stauffer VL, Wang S, Voulgaropoulos M, Skljarevski V, Kovacik A, Aurora SK (2019). Effect of Galcanezumab following treatment cessation in patients with migraine: results from 2 randomized phase 3 trials. Headache..

[50] de Hoon J, Van Hecken A, Vandermeulen C, Yan L, Smith B, Chen JS (2018). Phase I, randomized, double-blind, placebo-controlled, single-dose, and multiple-dose studies of Erenumab in healthy subjects and patients with migraine. Clin Pharmacol Ther.

[51] Fiedler-Kelly JB, Cohen-Barak O, Morris DN, Ludwig E, Rasamoelisolo M, Shen H, Levi M (2019). Population pharmacokinetic modelling and simulation of fremanezumab in healthy subjects and patients with migraine. Br J Clin Pharmacol.

[52] Kielbasa W, Quinlan T (2020). Population pharmacokinetics of Galcanezumab, an anti-CGRP antibody, following subcutaneous dosing to healthy individuals and patients with migraine. J Clin Pharmacol.

[53] Diamond S, Bigal ME, Silberstein S, Loder E, Reed M, Lipton RB (2007). Patterns of diagnosis and acute and preventive treatment for migraine in the United States: results from the American Migraine Prevalence and Prevention study. Headache..

